# 4D echocardiographic detection of early right atrial and ventricular dysfunction following dual-chamber pacing

**DOI:** 10.1186/s12872-025-05388-y

**Published:** 2025-12-05

**Authors:** Ganesh G Kamath, Krishnananda Nayak, Mukund A Prabhu, Sridevi Prabhu, Umesh Pai, Karthik A Naik

**Affiliations:** 1 Department of Allied Health Professions, Sikkim Manipal Institute of Medical Sciences, Sikkim Manipal University, Gangtok, Sikkim 737102 India; 2 Department of Cardiovascular Technology, Manipal College of Health Professions, Manipal Academy of Higher Education, Manipal, Karnataka 576104 India; 3 Department of Cardiology (Electrophysiology Division), Kasturba Medical College, Manipal Academy of Higher Education, Manipal, Karnataka 576104 India

**Keywords:** 4D echocardiography, Right atrial strain, Right ventricular function, Dual-chamber pacemaker, Tricuspid regurgitation

## Abstract

**Background:**

Although the influence of pacing on left ventricular function is well documented, the effect of right ventricular (RV) pacing, particularly concerning lead location (septal vs. apical), on RV function remains underexplored. Furthermore, there is a lack of literature regarding right atrial (RA) remodelling and functional changes following pacemaker implantation.

**Purpose:**

To assess the effect of a dual-chamber pacemaker on RA and RV structure and function, and their correlation with the site of RV lead positioning using 4D echocardiography.

**Methods:**

This prospective study evaluated 22 patients undergoing permanent dual-chamber pacemaker implantation. Comprehensive clinical and transthoracic echocardiographic assessments were performed at three time points: pre-implantation, pre-discharge, and at the 3-month follow-up. RV function was quantified using tricuspid annular plane systolic excursion, myocardial performance index, global longitudinal strain, and 3D RV ejection fraction. RA function was assessed for reservoir, conduit, and contractile phases using strain analysis. All echocardiographic measurements were conducted by a single echocardiographer utilizing the 4D Vivid S70N system. Device programming parameters were recorded postoperatively.

**Results:**

Although conventional parameters remained within the normal range, functionally, a decline in RA reservoir and conduit strain (*p* = 0.01 and *p* = 0.02, respectively) and RV GLS (*p* < 0.001) was observed. TR severity also worsened in nearly 40% of patients compared to baseline (*p* < 0.001). All patients had a pacing burden greater than 80%.

**Conclusion:**

RA and RV structural and functional changes begin within 3 months, irrespective of lead site, although non-septal sites exhibited a greater impairment.

## Introduction

Cardiac pacing remains a cornerstone in the management of symptomatic bradyarrhythmias, particularly sinus node dysfunction and advanced atrioventricular block. However, its long-term impact on cardiac mechanics, especially beyond the left ventricle, continues to raise clinical concerns [1]. Although right ventricular (RV) apical pacing is known to induce electromechanical dyssynchrony and left ventricular dysfunction, its influence on right heart structure and function is less well understood. A growing body of evidence links RV pacing with the development or worsening of tricuspid regurgitation (TR), likely due to mechanical interference with leaflet coaptation and altered hemodynamics [2]. Progressive TR, in turn, can adversely affect right atrial (RA) and RV function, predisposing patients to heart failure and arrhythmias [3]. Despite this clinical relevance, the effects of RV lead positioning, especially septal versus non-septal, on early RA and RV remodeling remain underexplored [4]. 

Traditional imaging tools, including fluoroscopy and 2D echocardiography, often lack the resolution to localize pacing leads precisely or detect subtle functional changes [5]. However, 4D echocardiography now allows real-time, volumetric assessment of RV and RA structure, strain-based functional analysis, and precise en-face visualization of the tricuspid valve and pacing leads. This study aimed to evaluate early structural and functional changes in the right heart following dual-chamber pacemaker implantation using advanced 4D echocardiography, and to assess whether these changes vary by RV lead position. We hypothesized that non-septal RV lead placement would be associated with more pronounced subclinical right heart dysfunction within the first 3 months post-implantation, even when conventional measures remain within normal limits [6, 7]. 

## Methodology

### Participant recruitment

This prospective, observational longitudinal study was conducted at a Kasturba hospital, Manipal, India, during April 2024 and February 2025. Adult patients referred for dual-chamber permanent pacemaker implantation for symptomatic bradyarrhythmias, such as sinus node dysfunction or high-grade atrioventricular block, were screened consecutively. The inclusion criteria comprised (1) age ≥ 18 years, (2) preserved left ventricular systolic function (LVEF ≥ 50%), and (3) a good acoustic window for transthoracic echocardiography. Patients with pre-existing moderate-to-severe tricuspid regurgitation (TR), congenital heart disease, previous cardiac surgery, or any contraindications to serial imaging were excluded. Of the 55 eligible participants, 22 underwent both baseline and follow-up echocardiographic evaluations and were included in the final analysis. The right ventricular lead position was classified as septal or non-septal based on 4D en-face visualization of the tricuspid valve annulus.

### Ethical approval

The research plan received approval from the Institutional Ethics Committee of Kasturba Medical College and Hospital, Manipal Academy of Higher Education (IEC approval no. IEC2 216/2024), and CTRI registration was done. All procedures complied with the Declaration of Helsinki, and written informed consent was obtained from all participants prior to recruitment.

### Cardiac assessment using 4D echocardiography

All patients underwent serial transthoracic echocardiographic evaluations at three predetermined time points: (1) pre-implantation (baseline), (2) pre-discharge (within 72 h of implantation), and (3) 3-month follow-up. Imaging was performed using a Vivid S70N ultrasound system (GE Healthcare, Chicago, IL, USA) and a 4Vc-D matrix-array transducer optimized for volumetric and speckle-tracking analysis. One echocardiographer conducted all acquisitions to discard inter-operator variability. Image optimization was performed in accordance with EACVI/ASE guidelines for chamber quantification.

### 3D volumetric measurement and global longitudinal strain

Right ventricular (RV) chamber geometry was measured using basal, mid, and distal diameters, RV outflow tract (RVOT) measurement, and tricuspid annular diameter from apical and parasternal windows. Real-time full-volume 3D datasets were acquired for volumetric reconstruction. RV end-diastolic volume (EDV), end-systolic volume (ESV), stroke volume, and ejection fraction (EF) were calculated using semi-automated 3D analysis software. Myocardial performance was described by RV fractional area change (FAC), TDI-derived systolic annular velocity (Sm), and RVOT velocity-time integral (VTI).

**Fig. 1:**
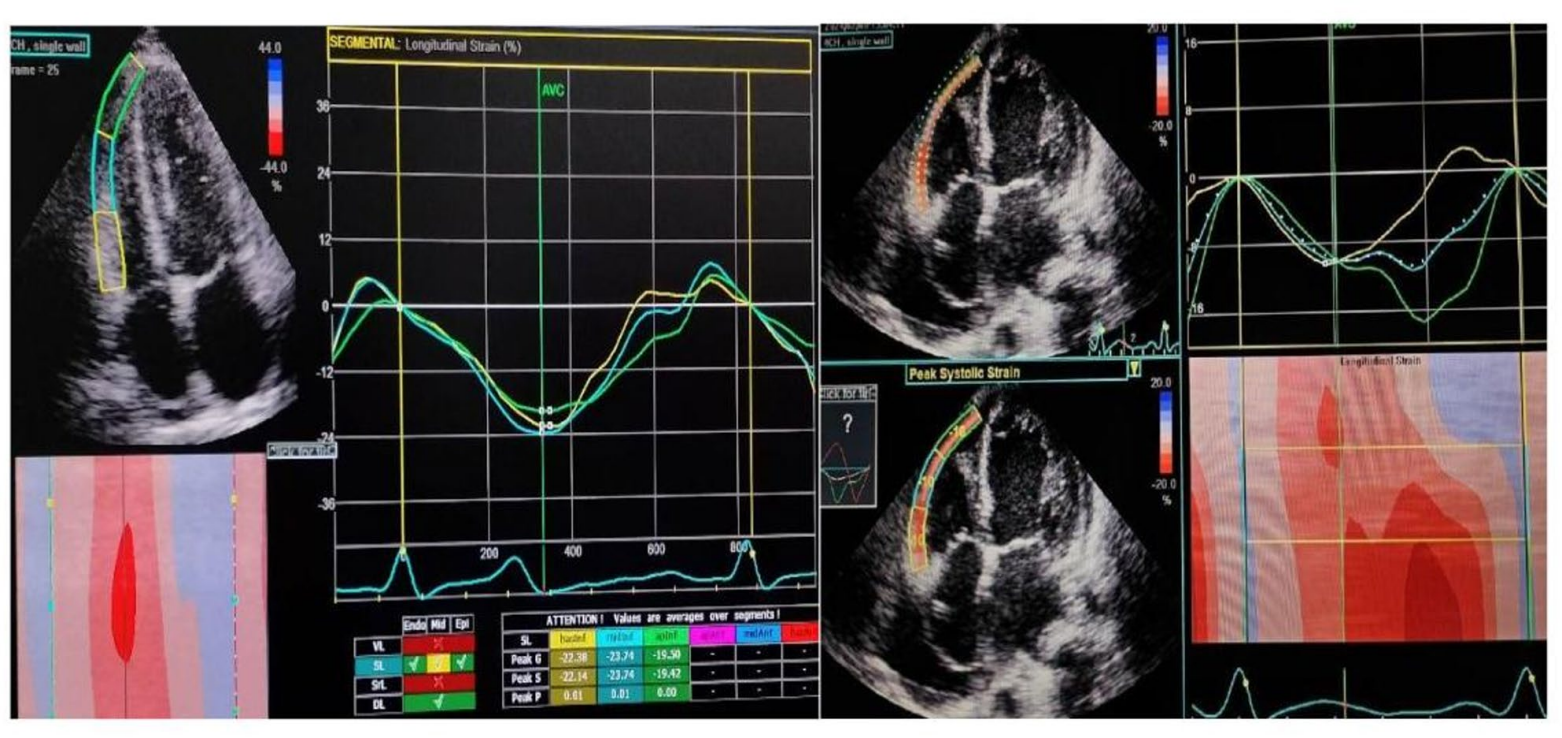
RV Free wall strain analysis using Echo PAC system

**Fig. 2:**
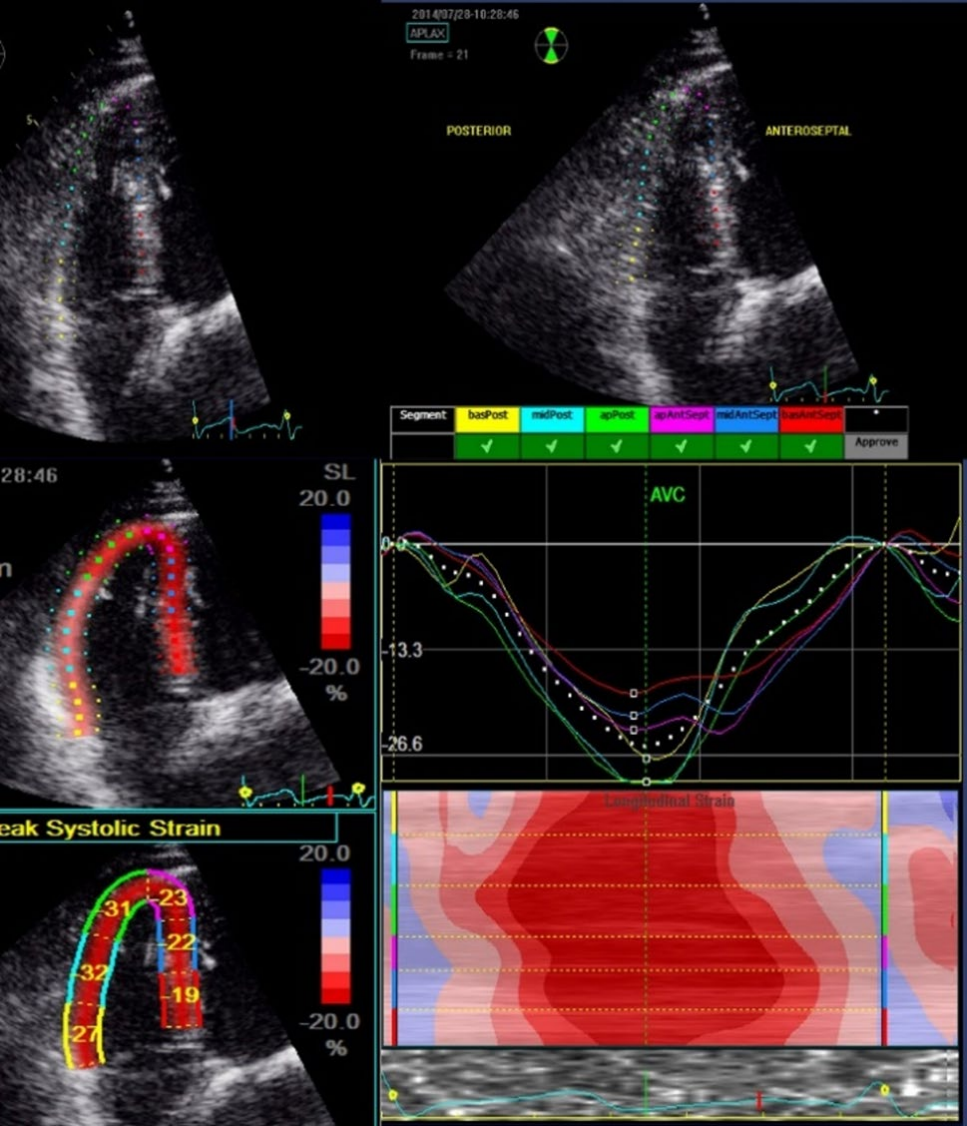
RV Global longitudinal strain using Echo PAC system

Advanced functional imaging employed 4D speckle-tracking echocardiography to derive RV global longitudinal strain (GLS) from basal, mid, and apical free wall segments in the RV-directed apical four-chamber view. Values were averaged to generate an average GLS index. Additionally, RV Free wall strain (FWS) analysis was done to eliminate the effect of LV Fig. 1 : RV Global longitudinal strain using Echo PAC system **(**Fig. 1, Fig. 2**)** Right atrial (RA) function was assessed through 3D volumetric measurements (EDV, ESV, ejection fraction) and phasic strain analysis, encompassing reservoir, conduit, and contractile phases. TR severity was quantified using a multiparametric integrative method according to ASE/EACVI guidelines, including color Doppler jet area, vena contracta width, continuous-wave Doppler morphology, and inferior vena cava (IVC) diameter and collapsibility index.

The lead position was determined by 4D en-face reconstruction of the tricuspid valve, and the lead course was classified as septal if it traversed the posterior aspect of the septal leaflet and non-septal if it crossed the free wall or commissural areas. All classifications were confirmed by two independent readers for consistency.

### Statistical analysis

All statistical analyses were performed using SPSS software (IBM Corp., Armonk, NY, USA). Continuous variables were tested for normality using the Shapiro–Wilk test and expressed as mean ± standard deviation or median (interquartile range), as appropriate. Categorical variables were presented as frequencies and percentages. A two-way repeated-measures ANOVA was used with time (baseline, post-implantation, 3 months) as the within-subject factor and RV lead position (septal vs. non-septal) as the between-subject factor. Bonferroni correction was applied to control Type I error for multiple pairwise comparison. A two-tailed p-value of < 0.05 was considered statistically significant.

## Results

### Description

**Fig. 3:**
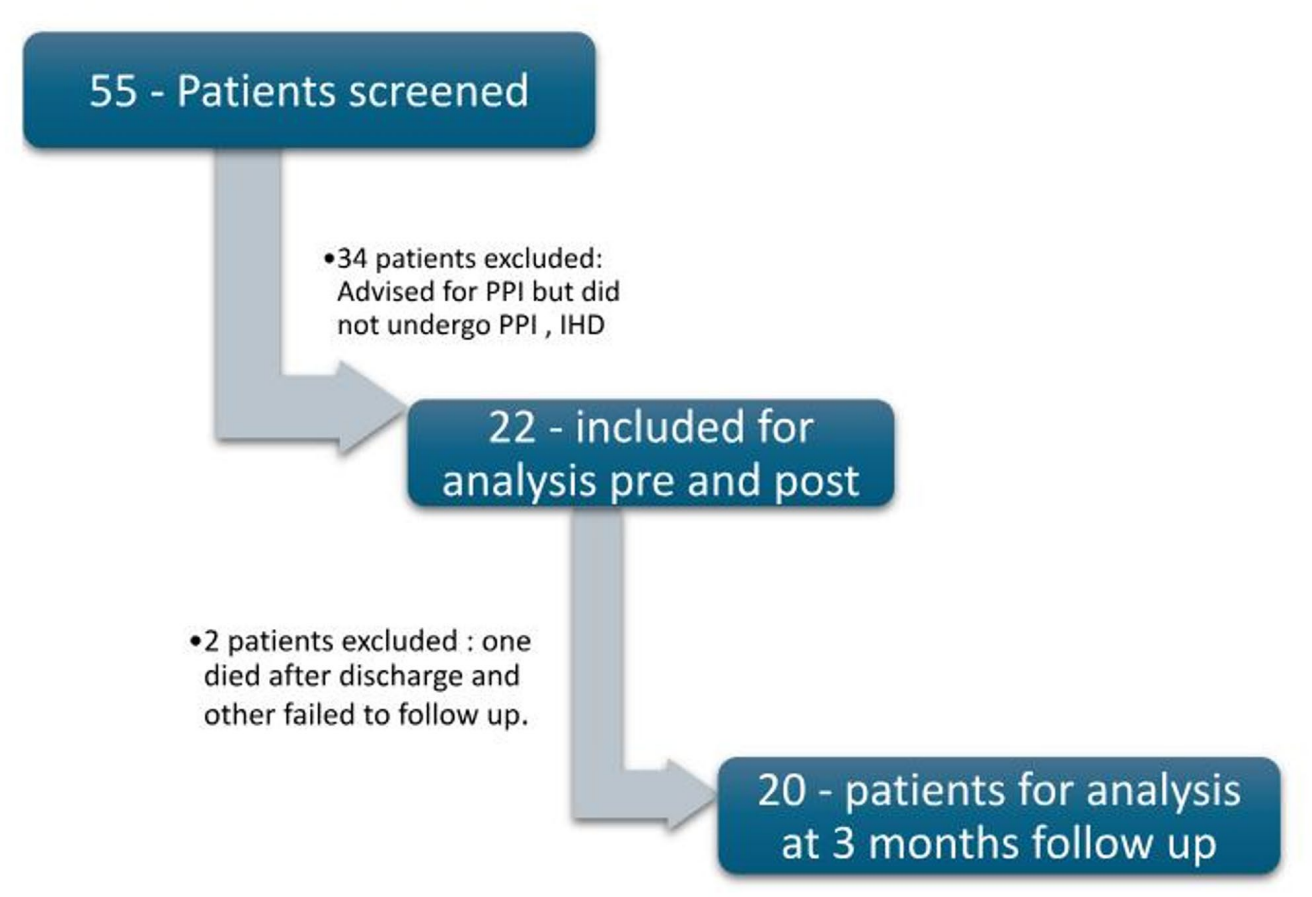
Study flow chart

The final study group consisted of 22 subjects with a relatively even sex distribution: 54.5% male (*n* = 12) and 45.5% female (*n* = 10). The mean age of the cohort was 68.8 ± 10.1 years. All patients had preserved left ventricular systolic function (LVEF ≥ 50%) at baseline and were implanted with dual-chamber pacemakers for symptomatic bradyarrhythmias. The mean pacing burden at follow-up was > 80% in all participants. During the study period, 2 patients were lost to follow-up at 3 months (Fig. 3).

### Right ventricular remodelling

Significant structural remodeling of the RV was observed over time. RV basal, mid, and distal dimensions decreased significantly from baseline to post-implantation and continued to decline at 3 months (*p* < 0.001 for all). RV end-diastolic volume (EDV) and end-systolic volume (ESV) showed a similar trend, with progressive volume reduction (EDV: 44.9 ± 2.8 mL to 31.6 ± 11.1 mL, *p* < 0.001; ESV: 25.3 ± 8.0 mL to 16.8 ± 5.9 mL, *p* < 0.001). Ejection fraction decreased significantly post-implantation (55.8 ± 12.1% to 41.0 ± 7.6%, *p* < 0.001), with partial recovery at 3 months (47.8 ± 12.5%, *p* = 0.001). Although conventional markers such as TAPSE and MPI remained within normal limits, a significant decline in RV strain was observed. Average RV GLS deteriorated from − 19.5 ± 2.9% at baseline to − 15.8 ± 3.3% at 3 months (*p* < 0.001), suggesting early RV dysfunction. To exclude the effects of LV on RV, we also perfomed RV FWS which showed similar trend (*p* < 0.001). RVTDI Sm and RVOT-VTI also declined significantly (*p* < 0.001), reflecting reduced systolic performance and forward flow (Table 1).

**Table 1:** RV parameters with time

Variable	Pre (Mean ± SD)	Post (Mean ± SD)	F/u at 3 months (Mean ± SD)	*p* value (Pre vs. post vs. f/u)	*P* value (Pre vs. Post)	*P* value (Pre vs. f/u)
Basal RV dimension (mm)	38.6 ± 5.7	33.7 ± 2.9	33.1 ± 5.1	**< 0.001**	**< 0.001**	**0.003**
Mid RV dimension (mm)	34.2 ± 5.4	28.3 ± 5.3	29.2 ± 7.5	**< 0.001**	**< 0.001**	**< 0.001**
Distal RV dimension (mm)	23.2 ± 5.2	19.2 ± 4.7	17.5 ± 4.8	**< 0.001**	**0.001**	**0.001**
RV dimension (mm)	71.9 ± 9.1	68.1 ± 9.1	69.2 ± 10.7	**0.011**	**0.007**	0.053
Proximal RVOT (mm)	21.8 ± 3.0	21.7 ± 1.8	19.7 ± 2.9	**< 0.001**	0.711	**< 0.001**
Distal RVOT (mm)	20.5 ± 2.2	21.68 ± 1.8	19.8 ± 2.9	**0.010**	**0.011**	**< 0.001**
Tricuspid annulus (mm)	30.3 ± 4.6	19.4 ± 2.8	27.6 ± 4.8	**0.030**	0.561	**0.008**
End diastolic area (mm^2^)	16.7 ± 2.6	14.8 ± 1.8	13.1 ± 2.1	**< 0.001**	**< 0.001**	**< 0.001**
End systolic area (mm^2^)	11.2 ± 2.5	9.9 ± 2.1	8.7 ± 1.8	**< 0.001**	**0.001**	**< 0.001**
End diastolic volume (ml)	44.9 ± 2.8	38 ± 4.9	31.6 ± 11.1	**< 0.001**	**< 0.001**	**< 0.001**
End systolic volume (ml)	25.3 ± 8.0	22.8 ± 5.1	16.8 ± 5.9	**< 0.001**	0.423	**< 0.001**
Ejection fraction (%)	55.8 ± 12.1	41 ± 7.6	47.8 ± 12.5	**< 0.001**	**< 0.001**	**0.001**
Fraction area change (%)	25.6 ± 6.9	22.7 ± 6.4	26.9 ± 6.8	0.371	0.205	0.473
Stroke volume (ml)	25.6 ± 9.4	13.7 ± 4.7	13.8 ± 7.4	**< 0.001**	**< 0.001**	**< 0.001**
TAPSE (mm)	20 ± 1.1	19.8 ± 1.8	19.2 ± 2.2	0.125	0.459	0.108
RV MPI	0.39 ± 0.03	0.44 ± 0.10	0.41 ± 0.11	0.218	**0.029**	0.445
TDI Sm (m/s)	0.14 ± 0.01	0.13 ± 0.23	0.10 ± 0.02	**< 0.001**	**0.006**	**< 0.001**
RVOT-VTI (cm)	17.8 ± 3.7	13.5 ± 3.3	14.1 ± 4.3	**< 0.001**	**< 0.001**	**< 0.001**
Basal RV GLS (%)	22.1 ± 4.3	17.2 ± 3.9	16.8 ± 5.3	**< 0.001**	**< 0.001**	**< 0.001**
Mid RV GLS (%)	23.6 ± 7.4	20.1 ± 7.1	21.7 ± 7.2	0.089	0.102	0.111
Distal RV GLS (%)	19.2 ± 5.7	16.2 ± 6.6	12.3 ± 6.5	**< 0.001**	**0.023**	**< 0.001**
RV GLS (avg, %)	19.5 ± 2.9	16.2 ± 3.1	15.8 ± 3.3	**< 0.001**	**< 0.001**	**< 0.001**
RV FWS (%)	18.8 ± 2.3	16.1 ± 2.9	15.7 ± 3	**< 0.001**	**< 0.001**	**< 0.001**
RVSP (mmHg)	24 ± 10.6	29.2 ± 7.5	27.8 ± 9.1	0.086	0.095	0.101
Pulmonary artery acceleration time (ms)	118.7 ± 12.1	115.7 ± 12.4	105.5 ± 10.9	**0.001**	0.561	**0.002**

### Right Atrial Remodeling

RA dimensions and volumes showed mild structural changes; however, functional decline was more pronounced. RA EDV and ESV decreased over time (*p* < 0.05), while RA ejection fraction declined from 41.7 ± 8.6% to 30.9 ± 11.3% at 3 months (*p* < 0.001). RA reservoir strain decreased significantly from − 23.3 ± 6.6% to − 18.5 ± 7.2% (*p* = 0.002), and conduit strain showed a marked decline (− 22.5 ± 8.2% to − 13.1 ± 11.3%, *p* < 0.001). Contractile strain remained relatively unchanged (Table 2).

**Table 2:** RA parameters with time

Variable	Pre (Mean ± SD)	Post (Mean ± SD)	F/u at 3 months (Mean ± SD)	*p* value (Pre vs. post vs. f/u)	*P* value (Pre vs. Post)	*P* value (Pre vs. f/u)
Major axis (mm)	47 ± 5.5	45.3 ± 5.6	45.3 ± 6.6	0.194	**0.001**	0.188
Minor axis (mm)	36.4 ± 7.9	33.9 ± 8.1	33.9 ± 8.1	**0.006**	**0.001**	**0.010**
End diastolic area (mm2)	14.3 ± 3.4	14.2 ± 2.7	14.2 ± 2.7	0.967	0.678	0.870
End systolic area (mm2)	10.5 ± 2.7	10.3 ± 2.3	11.3 ± 2.9	0.067	0.495	0.164
End diastolic volume (ml)	43.3 ± 14.1	36.9 ± 14.5	34.8 ± 13.2	**0.021**	**0.004**	**0.024**
End systolic volume (ml)	28.2 ± 9.1	24.5 ± 9.1	22.5 ± 12	**0.004**	**0.002**	**0.004**
Ejection fraction (%)	41.7 ± 8.6	33.6 ± 11.6	30.9 ± 11.3	**< 0.001**	**< 0.001**	**< 0.001**
Conduit strain (%)	22.5 ± 8.2	13.5 ± 2.9	13.1 ± 11.3	**< 0.001**	**< 0.001**	**< 0.001**
Contractile strain (%)	9.8 ± 6.2	4.2 ± 2.4	10 ± 4.9	0.876	0.659	0.941
Reservoir strain (%)	23.3 ± 6.6	19.1 ± 6.3	18.5 ± 7.2	**< 0.001**	**0.002**	**0.002**

### Tricuspid regurgitation progression

Worsening TR severity was observed in a substantial subset of patients. At baseline, the majority had trivial or mild TR. By 3-month follow-up, moderate TR had developed in 40% of patients (*p* < 0.001). Both vena contracta width and TR jet area increased significantly over time.

Left ventricular ejection fraction (LVEF) reduced from 64.2% at baseline to 60.2% after implantation and 57.4% at 3 months (*p* < 0.001). Early fall was significant (*p* = 0.032), with further reduction by 3 months (*p* < 0.001). This indicates a marked deterioration in LV systolic function, possibly due to pacing-induced ventricular dyssynchrony.

### Subdivision of the study group into septal and non-septal lead categories

Among the 22 patients studied in total, the lead position as seen on 4D echocardiography in the tricuspid valve en face view was classified as septal in 13 patients (59.1%) and non-septal in 9 patients (40.9%). This categorization was according to the anatomic course of the pacing lead in association with the tricuspid valve annulus and leaflets. Leads touching the interventricular septum close to the septal leaflet or anterior commissure were considered septal; leads positioned over the posterior or free-wall commissural areas were classified as non-septal. These two groups were then utilized for further comparison of the severity of tricuspid regurgitation and concomitant right heart changes between study time points and serve as a Hypothesis generating for future studies.

Non-septal lead placement was associated with greater reductions in RV FWS GLS and more severe TR at follow-up, although statistical significance was limited by sample size. RA volumes and strain parameters also showed a trend toward greater functional decline in the non-septal group (Tables 3, 4, 5 and 6).

**Table 3:** Comparative analysis of TR severity in septal vs. non-septal lead positions

TR severity		Pre Frequency (%)	Post Frequency (%)	F/u at 3 months Frequency (%)
Trivial	*Septal*	4 (30.8)	0	0
	*Non-Septal*	4 (44.4)	1 (11.5)	1 (12.5)
Mild	*Septal*	4 (44.4)	5 (38.5)	4 (33.3)
	*Non-Septal*	9 (69.2)	7 (77.8)	6 (75)
Moderate	*Septal*	0	7 (53.8)	7 (58.3)
	*Non-Septal*	1 (11.1)	1 (11.1)	1 (12.5)
Moderate-Severe	*Septal*	0	1 (7.7)	1 (8.3)
	*Non-Septal*	0	0	0
	***p value***	0.321	0.097	0.096

**Table 4:** Comparison of subgroups with RV parameters

Variable	Septal			Non-Septal					
	**Pre (Mean ± SD)**	**Post (Mean ± SD)**	**F/u at 3 months (Mean ± SD)**	**Pre (Mean ± SD)**	**Post (Mean ± SD)**	**F/u at 3 months (Mean ± SD)**		p 1*	**p 2** ^**#**^
Basal RV dimension (mm)	38.7 ± 5.4	34.4 ± 3.02	35.2 ± 4.9	38.4 ± 6.6	32.7 ± 2.7	29.8 ± 3.6		**0.006**	0.100
Mid RV dimension (mm)	35.1 ± 5.6	29.5 ± 6.1	30.4 ± 7.7	33 ± 5.1	26.5 ± 3.6	27.4 ± 7.2		**<0.001**	0.859
Distal RV dimension (mm)	23.8 ± 5.1	19.1 ± 4.6	15.8 ± 4.1	22.3 ± 5.6	19.5 ± 5.4	15.8 ± 4.1		**<0.001**	0.328
RV dimension (mm)	73.8 ± 8.1	71.8 ± 4.5	71.6 ± 8	69.2 ± 10.3	62.5 ± 11.6	65.6 ± 13.5		**0.004**	0.151
Proximal RVOT (mm)	22.5 ± 3.5	22.3 ± 1.8	20.4 ± 3.3	20.6 ± 1.5	20.7 ± 1.6	18.4 ± 1.8		**<0.001**	0.851
Distal RVOT (mm)	20.4 ± 2.2	19.5 ± 2.8	19.5 ± 1.4	20.6 ± 1.5	20.7 ± 1.6	18.4 ± 2.5		**0.006**	0.423
Tricuspid annulus (mm)	30.4 ± 4.8	30.5 ± 5.1	29.8 ± 5.1	30.1 ± 4.4	30.5 ± 5.1	26.7 ± 4.4		**0.002**	0.298
End diastolic area (mm^2^)	16.4 ± 2.5	14.7 ± 1.5	13.1 ± 1.5	17.2 ± 2.9	15.4 ± 9.3	13.1 ± 2.9		**<0.001**	0.907
End systolic area (mm ^2^)	10.7 ± 2.1	9.9 ± 2.7	8.2 ± 1.7	11.8 ± 2.9	9.9 ± 1.9	8.2 ± 1.7		**<0.001**	0.094
End diastolic volume (ml)	45.2 ± 2.4	37.3 ± 4.2	31.1 ± 11	44.5 ± 3.6	39 ± 6.1	32.4 ± 11.9		**<0.001**	0.809
End systolic volume (ml)	25.8 ± 8.7	22.8 ± 5.8	17 ± 6.8	24.5 ± 7.3	22.7 ± 3.8	17 ± 6.8		**<0.001**	0.897
Ejection fraction (%)	56.8 ± 14	46.6 ± 11.5	42.1 ± 8.7	54.4 ± 9.9	49.6 ± 14.4	39.9 ± 5.8		**0.03**	0.215
Fraction area change (%)	24.5 ± 2.5	26.6 ± 5.4	24.8 ± 4.3	27.3 ± 10.3	29.4 ± 7.8	30 ± 9		0.383	0.072
Stroke volume (ml)	24.6 ± 9.6	12.8 ± 3.5	13.7 ± 8.4	27.3 ± 9.6	15.1 ± 6.1	14 ± 6.5		**<0.001**	0.646
TDI Sm (m/s)	0.14 ± 0.01	0.13 ± 0.02	0.1 ± 0.2	0.13 ± 0.02	0.12 ± 0.02	0.11 ± 0.02		**<0.001**	**0.018**
RVOT-VTI (cm)	17.8 ± 3.8	13.3 ± 3.1	12.9 ± 3.4	17.9 ± 3.9	13.7 ± 3.1	15.7 ± 5.2		**<0.001**	0.646
Basal RV GLS (%)	21.9 ± 4.5	16.2 ± 4.3	16.7 ± 6.3	22.7 ± 4.3	18.7 ± 2.9	16.7 ± 4.1		**<0.001**	0.577
Mid RV GLS (%)	23.9 ± 7.7	22.8 ± 8	20.5 ± 4.7	23.1 ± 7.5	18.1 ± 4.9	20.5 ± 4.7		0.176	0.349
Distal RV GLS (%)	19.6 ± 5.1	17 ± 7.2	12.6 ± 6.5	18.6 ± 6.8	14.8 ± 5.9	11.8 ± 5.9		**<0.001**	0.843
RV GLS (avg, %)	18.8 ± 3.1	15.8 ± 3.2	16.2 ± 2.9	20.4 ± 2.5	16.8 ± 2.7	16.2 ± 2.9		**<0.001**	0.577
RV FWS (%)	18.2 ± 2.6	15.8 ± 3.1	15.4 ± 3.3	19.5 ± 1.9	16.6 ± 2.5	16.3 ± 2.7		**<0.001**	0.894

*p1 = p value when compared across timepoints

^#^p2 = p value when compared among subgroups

**Table 5:** Comparison of subgroups with RA parameters

Variable	Septal			Non-Septal					
	Pre (Mean ± SD)	Post (Mean ± SD)	F/u at 3 months (Mean ± SD)	Pre (Mean ± SD)	Post (Mean ± SD)	F/u at 3 months (Mean ± SD)		p 1*	p 2 ^#^
Major axis (mm)	47.2 ± 6.5	45.5 ± 7.8	45.6 ± 9.3	47.3 ± 6.5	45 ± 4.9	44.5 ± 7.2		0.216	0.916
Minor axis (mm)	37.8 ± 7.6	35.3 ± 7.8	34.7 ± 10.5	34.3 ± 8.4	31.8 ± 8.3	29.3 ± 8.3		**0.006**	0.632
End diastolic area (mm2)	14.7 ± 3.5	14.5 ± 2.9	14.9 ± 2.6	13.7 ± 3.4	13.7 ± 2.4	13.1 ± 2.4		0.940	0.586
End systolic area (mm2)	10.8 ± 2.7	10.5 ± 2.3	11.9 ± 2.7	10.1 ± 3.1	10.1 ± 3.1	10.3 ± 2.9		0.129	0.329
End diastolic volume (ml)	44.3 ± 15.6	38.7 ± 14.1	37.9 ± 13.7	41.6 ± 12.2	34 ± 15.7	29.6 ± 11		**0.018**	0.673
End systolic volume (ml)	30.3 ± 9.5	26.6 ± 10.2	25.4 ± 13.6	25 ± 7.8	21.2 ± 6.5	18.3 ± 8.1		**0.005**	0.820
Ejection fraction (%)	39.1 ± 6.6	32.6 ± 10.2	30.6 ± 10.4	45.6 ± 10.2	35.1 ± 13.9	31.3 ± 13.2		**<0.001**	0.272
Conduit strain (%)	20.4 ± 7.5	13.2 ± 2.5	12.6 ± 5.8	25.5 ± 8.5	13.8 ± 2.5	13.6 ± 5.2		**<0.001**	0.340
Contractilestrain (%)	11 ± 5.4	8 ± 3.6	9.3 ± 4.5	8.1 ± 7.3	10.8 ± 6.2	11 ± 5.7		0.903	0.209
Reservoir strain (%)	23.4 ± 6.32	18.7 ± 7.2	18.7 ± 8.6	23.1 ± 7.6	19.6 ± 5.4	18.1 ± 5.1		**<0.001**	0.800

*p1 = p value when compared across timepoints

#p2 = p value when compared among subgroups

**Table 6:** Comparison of subgroups with additional parameters

Variable	Septal			Non-Septal					
	Pre (Mean ± SD)	Post (Mean ± SD)	F/u at 3 months (Mean ± SD)	Pre (Mean ± SD)	Post (Mean ± SD)		F/u at 3 months (Mean ± SD)	p 1*	p 2 ^#^
RVSP (mmHg)	23.5 ± 8.7	25.9 ± 7	27.8 ± 11.9	24.7 ± 13.8	34.4 ± 5.2		27.7 ± 6.4	**0.047**	0.155
PAAT (ms)	123.9 ± 8.9	119.2 ± 9.1	103.8 ± 12.3	110.6 ± 12.7	110 ± 15.3		109 ± 8.2	**0.004**	**0.013**
TR jet area (mm)	1.2 ± 1.1	3.8 ± 2.5	4.7 ± 2.4	1.1 ± 1.2	1.4 ± 1.1		1.9 ± 1.4	**< 0.001**	**0.017**
TR vena contracta (mm)	1.1 ± 0.11	3.6 ± 3.1	4.1 ± 3.1	1 ± 0.00	1.2 ± 0.2		1.9 ± 1.4	**0.009**	0.118
LV-EF (%)	64.5 ± 9.1	58.9 ± 5.2	54.7 ± 6.8	63.8 ± 0.8	62.1 ± 2.6		61.4 ± 5.7	**0.003**	0.093

*p1 = p value when compared across time points

p2 = p value when compared among subgroups

### Post-procedure complications and mortality

Post-procedural complications were noted in two patients (22.2%). In the septal group, one patient (7.7%) developed complications due to pacemaker-associated pericarditis. Whereas in non-septal group, one patient died during the follow-up period; and the event was considered unrelated to pacemaker implantation.

## Discussion

This study demonstrates that structural and functional changes in the right heart begin as early as 3 months following permanent pacemaker implantation, even in patients with preserved left ventricular systolic function. Significant reductions in right atrial (RA) reservoir and conduit strain, right ventricular (RV) global longitudinal strain (GLS), and progressive tricuspid regurgitation (TR) were observed. These findings indicate that early, subclinical dysfunction of the right heart occurs post-pacing, detectable only through advanced imaging modalities like 4D echocardiography.

While most previous studies have focused on pacing-induced left ventricular dysfunction, the right heart has often been under-recognized [8–10]. Our findings add to growing evidence that pacing also affects right-sided chambers. The decline in RA reservoir and conduit strain indicates impaired atrial compliance and altered filling pressures, even before visible chamber enlargement [11]. RA strain, especially the reservoir phase, is a sensitive marker of volume loading and right atrial performance. These changes occurred despite patients being in sinus rhythm and having normal chamber dimensions.

Similarly, RV function, assessed using 4D GLS, showed early decline. Although parameters like TAPSE and MPI remained within normal limits, RV strain worsened significantly, indicating early systolic dysfunction. Prior studies have shown that pacing can impair RV mechanics, especially in the setting of altered septal motion or increased afterload [11, 12]. The temporal changes are likely to be reflective of restoration of AV synchrony and resultant normalisation of filling pressures, reduced preload rather than remodelling. The aggravation of the systolic measures likely is a long-term consequence of pacing and would need longer follow up to reveal itself. Abdelgawad et al. observed significant deterioration in RV strain indices despite preserved conventional echocardiographic parameters, reinforcing that strain imaging provides greater sensitivity in detecting early dysfunction [11]. 

Progressive TR was another consistent finding, seen in nearly 40% of our patients at 3 months. Though mild in most cases, the direction of change was clear. TR progression may result from direct lead interference with leaflet coaptation or altered subvalvular tension, both contributing to mechanical regurgitation [12–14]. These mechanical effects, in turn, contribute to volume overload and declining RA/RV strain.

Although our study was not primarily designed to assess lead position as a primary outcome, we observed a trend toward greater impairment in patients with non-septal lead placement. However, this remains a secondary observation and requires further evaluation in larger cohorts [15, 16]. 

The secondary observation in our cohort was a reduction in LVEF which may indicate an early ventricular dysfunction associated with pacing-induced dyssynchrony. This finding is in line with previous reports of similar modest LVEF decline in patients with normal baseline LVEF and points out the need for long-term follow-up of LV function [17–19]. 

Early reductions in RA and RV strain seen in our study may represent subclinical remodelling that precedes pacing-induced cardiomyopathy or future heart failure progression. Recent findings have demonstrated that impaired RA strain, particularly RA contractile strain is predictive of atrial fibrillation [20]. In our study, RA reservoir and conduit strain also deteriorated after pacemaker implantation. These results put together suggest that the pacing-related dysfunction of the right atrium may increase the risk of future AF which leads to stroke and provide support for the routine screening of RA strain in order to identify early atrial myopathy.

Although the long-term consequences of diminished RA and RV strain need further clarification, our data indicate the possible value in implementing 4D echocardiography in post-implantation follow-up. Early strain measurement, particularly in patients with elevated pacing burden or non-septal lead sites, could be included by clinicians in complete pacing surveillance, but this needs to be confirmed.

We recognize that our study is hypothesis-generating. It needs to be followed by larger, long-term follow-up studies correlated with outcomes to determine diagnostic thresholds.

### Limitation

The primary limitation of this study is the small sample size due to strict inclusion criteria of requiring quality 4D datasets at all three time points. Subgroup comparisons of septal vs. non-septal lead positions should be viewed as exploratory and serves as a hypothesis generator for a larger population. Third, Longer follow-up is needed to reveal the long-term effects of pacing on cardiac function. Lastly, while 4D echocardiographic analysis is certainly advanced, it is susceptible to image quality, acoustic access. However, all acquisitions were performed by one experienced operator to reduce inter-operator variability.

## Conclusion

In patients with permanent pacemaker implantation, right ventricular structural and functional changes can commence within a short term, involving progressive deterioration in strain and tricuspid regurgitation. These changes were observed regardless of lead site, although non-septal sites showed a trend toward slightly greater impairment. While ejection fraction was generally preserved, more advanced measurements like 4D strain and RV volumes revealed subclinical dysfunction at an early stage.

## Data Availability

The data that supports the findings of this study are available upon request from the corresponding author. Due to the sensitive nature of the data and to ensure participant privacy, the data cannot be publicly shared or deposited in a public repository. The research team will review requests for data access to ensure compliance with ethical and legal requirements.

## Abbreviations

AF: Atrial fibrillation
ANOVA: Analysis of variance
ASE: American Society of Echocardiography
AV: Atrioventricular
CTRI: Clinical Trials Registry of India
EACVI: European Association of Cardiovascular Imaging
EDV: End-diastolic volume
EF: Ejection fraction
ESV: End-systolic volume
FAC: Fractional area change
IVC: Inferior vena cava
LV: Left ventricle
PPI: Permanent Pacemaker implantation
RA: Right atrium
RV: Right ventricle
RVOT: Right ventricular outflow tract
RVTDI: Right ventricular tissue Doppler imaging
SPSS: Statistical Package for Social Sciences
TAPSE: Tricuspid annular plane systolic excursion
TDI: Tissue Doppler imaging
TR: Tricuspid regurgitation
VTI: Velocity time integral
